# Contemporary Outcomes of Neonatal Exomphalos: A 12‐Year Single‐Centre Experience

**DOI:** 10.1111/ped.70580

**Published:** 2026-10-22

**Authors:** Agana Gnanakuruparan, Chandrasen K. Sinha, Sandeep Shetty

**Affiliations:** ^1^ City St George's University of London London UK; ^2^ Neonatal Intensive Care Unit St George's Hospital NHS Foundation Trust London UK; ^3^ Department of Women and Children's Health King's College London London UK

**Keywords:** congenital anomalies, exomphalos, exomphalos major, neonatal surgery, omphalocele

## Abstract

**Aim:**

Exomphalos is a congenital abdominal wall defect associated with significant neonatal morbidity and mortality. Contemporary UK outcome data remain sparse. This study describes outcomes, anomalies, and management strategies for live‐born infants with exomphalos over a 12‐year period at a UK tertiary neonatal surgical centre.

**Methods:**

A retrospective audit of 36 live‐born infants admitted between 2011 and 2023 was performed. Infants were classified as exomphalos major (ExoMaj; defect ≥ 5 cm and/or containing the liver) or exomphalos minor (ExoMin; defect < 5 cm). Outcomes included mortality, hospital stay, ventilation duration, age to full enteral feeds, and complication rates.

**Results:**

Of 36 infants, 53% (*n* = 19) had ExoMaj and 47% (*n* = 17) had ExoMin. Overall mortality was 11% (*n* = 4), all deaths in the ExoMaj group (21% vs. 0%; *p* = 0.04). ExoMaj was associated with longer hospital stay (26 vs. 8 days; *p* < 0.001), longer ventilation (2 vs. 1 days; *p* = 0.03), higher complication rates (70% vs. 0%; *p* = 0.004), and delayed full enteral feeds (20 vs. 7 days; *p* = 0.003). All deaths were attributable to respiratory failure secondary to pulmonary hypertension and/or pulmonary hypoplasia. All ExoMaj infants with PPHN and/or pulmonary hypoplasia died, whilst the infant with pulmonary stenosis survived. Pulmonary defects predominated in ExoMaj (37%); Beckwith‐Wiedemann syndrome in ExoMin (41%).

**Conclusions:**

All mortality occurred in ExoMaj and was attributable exclusively to PPHN and/or pulmonary hypoplasia. Anomaly type may be more clinically relevant than anomaly presence alone. These hypothesis‐generating findings have relevance to antenatal counseling. Larger multicentre studies are required to define independent predictors of outcome.

## 1 Introduction

Exomphalos is a congenital midline anterior abdominal wall defect occurring in approximately 1 in 5000 live births, characterized by herniation of intra‐abdominal organs through the umbilical ring, covered by a membrane of peritoneum and amnion [1]. The condition is classified according to defect size and contents: exomphalos major (ExoMaj) is defined as a defect ≥ 5 cm in diameter and/or containing the liver, whilst exomphalos minor (ExoMin) is defined as a defect < 5 cm [1].

Existing literature consistently reports superior survival in ExoMin compared with ExoMaj [2]. A landmark study by Vachharajani et al. [3] demonstrated higher mortality rates, prolonged time to full enteral feeds, and greater complication rates in ExoMaj neonates, whilst cautioning that ExoMin neonates are not without risk and require vigilant follow‐up [3].

Up to 75% of exomphalos neonates have concomitant associated anomalies—including chromosomal abnormalities, malformation syndromes, and structural defects—independently linked to worse outcomes [1]. However, data on the specific incidence and pattern of these anomalies, and their influence on clinical outcomes, remain limited, hampering reliable prognostication and family counseling.

This retrospective audit aims to characterize outcomes, associated anomalies, and management strategies for live‐born ExoMaj and ExoMin neonates over a 12‐year period at a UK tertiary neonatal unit. To our knowledge, this represents one of the largest contemporary UK single‐centre datasets for this condition. In particular, we describe the pattern of associated anomalies by classification, the role of pulmonary complications in determining mortality, and the outcomes of conservative management—areas where UK‐specific data remain limited.

## 2 Methods

### 2.1 Patient Population

All live‐born infants with a diagnosis of exomphalos admitted to the Neonatal Unit at St George's Hospital NHS Foundation Trust between 1st January 2011 and 30th September 2023 were retrospectively reviewed following local audit approval (Audit registration number‐AUDI005026). Infants were identified from the neonatal electronic database (Badgernet) and contemporaneous medical records. Variables collected included gender, gestational age, birthweight, mode of delivery, and type of exomphalos.

Patients were classified as: (a) ExoMaj (defect ≥ 5 cm) or (b) ExoMin (defect < 5 cm). The cohort was also classified as isolated or non‐isolated exomphalos. Non‐isolated exomphalos comprised infants with any of the following associated anomalies:

- Cardiac: patent ductus arteriosus (PDA), ventricular septal defect (VSD), or atrial septal defect (ASD). Cardiac lesions were defined according to contemporaneous echocardiographic reports documented by the treating cardiology team.
- Pulmonary: persistent pulmonary hypertension of the newborn (PPHN), pulmonary hypoplasia, and pulmonary stenosis.
- Syndromic: Beckwith‐Wiedemann syndrome.
- 6p chromosomal deletion.
- Other: inguinal hernia, spina bifida, talipes equinovarus, talipes calcaneovalgus, congenital lymphoedema, craniosynostosis, renal dysplasia, bladder exstrophy with epispadias, bilateral retractile testes, undescended testicles, anal atresia.

### 2.2 Postnatal Outcomes

Outcomes of interest included postnatal death, length of hospital stay, days on ventilation (invasive and non‐invasive), age to achieve full enteral feeds, postoperative complications (wound infection—either culture‐positive or documented purulence), bacteraemia (confirmed by positive blood cultures), and duration of follow‐up.

### 2.3 Management

Conservative management involved Medihoney (Derma Sciences, Trenton, NJ) or Flamazine (Smith & Nephew, London, UK) (silver sulfadiazine) dressings changed every 24–48 h. Surgical treatment comprised primary closure or staged closure via silo. Primary skin closure was performed with or without fascial closure. Staged closure involved surgical silo insertion permitting serial visceral reduction, followed by primary skin closure with or without fascial repair once the exomphalos was reducible. Silo insertion was defined as the first surgical intervention.

### 2.4 Statistical Analysis

Statistical analysis was performed using IBM SPSS version 22 (IBM Corporation, Armonk, NY). Mann–Whitney U tests were applied for continuous variables and chi‐square analysis for categorical variables. A *p* value of ≤ 0.05 was considered statistically significant. Data are presented as median (range) unless otherwise stated. Fisher's exact test was used where expected cell counts were fewer than 5. Given the exploratory nature of this study and the limited sample size, adjustment for multiple comparisons was not performed.

## 3 Results

### 3.1 Overall Population

Over the 12‐year period, 36 live‐born infants with exomphalos were identified: 53% (*n* = 19) ExoMaj and 47% (*n* = 17) ExoMin. All had received a prenatal diagnosis. Ninety‐two per cent (*n* = 33) were delivered at the study institution and 8% (*n* = 3) transferred within 4 days of life. Fifty per cent (*n* = 18) were delivered vaginally, 36% (*n* = 13) by emergency caesarean section, and 14% (*n* = 5) by elective caesarean section. Associated congenital anomalies were present in 67% (*n* = 24) of infants. There were no confirmed cases of bacteraemia. Four infants died, giving an overall mortality of 11%. Median follow‐up was 4.2 years (range 3 months–11 years). Baseline characteristics and outcome measures are presented in Table 1.

**TABLE 1:** Infant characteristics and outcome measures: Exomphalos Major vs. Minor.

Variable	ExoMaj (*n* = 19) % or median (range)	ExoMin (*n* = 17) % or median (range)	Total (*n* = 36)	*p*
Gender				
Male	7/19 (37)	10/17 (59)	17/36 (47)	0.18
Female	12/19 (63)	7/17 (41)	19/36 (53)	
Associated anomalies	12/19 (63)	12/17 (71)	24/36 (67)	0.63
Mode of delivery				
Vaginal	9/19 (47)	9/17 (53)	18/36 (50)	0.73
Elective caesarean	3/19 (16)	2/17 (12)	5/36 (14)	0.72
Emergency caesarean	7/19 (37)	6/17 (35)	13/36 (36)	0.92
Gestational age (weeks)	37 (30–39)	38 (28–39)	—	0.22
Birthweight (g)	2795 (1245–3555)	3395 (1210–4770)	—	0.005
Hospital stay (days)	26 (2–65)	8 (3–16)	—	< 0.001
Ventilation duration (days)	2 (0–65)	1 (0–4)	—	0.03
Bacteraemia	0	0	—	—
Deaths	4/19 (21)	0 (0)	4/36 (11)	0.04

*Note:* Data are presented as median (range) for continuous variables and *n* (%) for categorical variables. Continuous variables were compared using the Mann–Whitney *U* test; categorical variables using chi‐square or Fisher's exact test, as appropriate.

### 3.2 Outcomes: ExoMaj Vs. ExoMin

There were no significant differences between groups in gender (*p* = 0.18), mode of delivery, gestational age (37 vs. 38 weeks; *p* = 0.22), or incidence of associated anomalies (63% vs. 71%; *p* = 0.63). Birthweight was significantly higher in ExoMin neonates (median 3395 vs. 2795 g; *p* = 0.005). ExoMaj was associated with longer hospital stay (26 vs. 8 days; *p* < 0.001) and longer duration of ventilation (2 vs. 1 days; *p* = 0.03). All observed mortality occurred among infants with ExoMaj (21% vs. 0%; *p* = 0.04), with ExoMin achieving 100% survival. ExoMaj neonates also demonstrated lower birthweights, possibly reflecting altered fetal growth patterns previously described in severe exomphalos [1], although this study was not designed to investigate causality.

All four deaths in ExoMaj were attributable to respiratory failure secondary to PPHN and/or pulmonary hypoplasia (Table 2). The most common associated anomaly in ExoMaj was pulmonary defects (37%), particularly PPHN and pulmonary hypoplasia, whilst Beckwith‐Wiedemann syndrome predominated in ExoMin (41%).

**TABLE 2:** Pulmonary defect type, management, and outcomes in the four neonates who died (all ExoMaj).

Neonate	Pulmonary defect	Hospital stay (days)	Ventilation (days)	Management	Age at death (days)
1	PPHN	26	26	Conservative	74
2	PPHN	65	65	Primary closure	64
3	Pulmonary hypoplasia	2	2	Conservative	1
4	PPHN and pulmonary hypoplasia	42	40	Conservative	42

*Note:* All deaths were attributable to respiratory failure. All four neonates had ExoMaj.

Abbreviation: PPHN, persistent pulmonary hypertension of the newborn.

### 3.3 Isolated vs. Non‐Isolated Exomphalos

No statistically significant differences were detected between isolated and non‐isolated exomphalos in terms of ExoMaj/ExoMin distribution, length of hospital stay, age at full enteral feed, postoperative complication rate, or mortality (Table 3), although the study was not powered to exclude clinically important differences. However, non‐isolated infants required significantly longer ventilation than isolated infants (2 vs. 0 days; *p* = 0.007).

**TABLE 3:** Outcomes by isolated vs. non‐isolated exomphalos.

Variable	Isolated (*n* = 12)	Non‐isolated (*n* = 24)	*p*
Hospital stay (days)	19.5 (5–39)	13.0 (2–65)	0.650
Age at full feeds (days)	13.5 (3–38)	9.5 (2–38)	0.953
Ventilation (days)	0 (0–5)	2 (0–65)	0.007
Mortality	0/12 (0%)	4/24 (17%)	0.278
Complications	1/12 (8%)	5/24 (21%)	0.640

*Note:* Data are presented as median (range).

Abbreviations: BW, birthweight; GA, gestational age; PPHN, persistent pulmonary hypertension of the newborn; Vent, ventilation.

### 3.4 Surgical Management and Outcomes

Eight ExoMaj neonates underwent primary closure and two underwent staged closure with a silo. All 17 ExoMin neonates underwent primary closure within 8 days of life. One ExoMaj neonate who underwent primary closure died postoperatively (age at death 64 days; cause: respiratory failure) and was excluded from the age at full enteral feed analysis. Of the 8 conservatively managed ExoMaj neonates, two died (ages at death: 74 and 40 days; both from respiratory failure secondary to PPHN/pulmonary hypoplasia) and were likewise excluded from that analysis. Surgical and conservative outcomes are summarized in Table 4 and (Figure 1).

**TABLE 4:** ExoMaj infants with pulmonary conditions: Survivors vs. non‐survivors.

	GA (weeks)	BW (g)	Pulmonary diagnosis	Ventilation (days)	Outcome
Non‐survivors (*n* = 4)					
Case 1	33	1245	PPHN	26	Died
Case 2	32	1500	PPHN + pulmonary hypoplasia	65	Died
Case 3	32	1500	Pulmonary hypoplasia	2	Died
Case 4	38	3400	PPHN + pulmonary hypoplasia	41	Died
Survivor (*n* = 1)					
Case 5	36	2225	Pulmonary stenosis only	2	Survived

*Note:* Data anonymised.

Abbreviations: BW, birthweight; GA, gestational age; PPHN, persistent pulmonary hypertension of the newborn; Ventilation, duration of ventilatory support.

**FIGURE 1:**
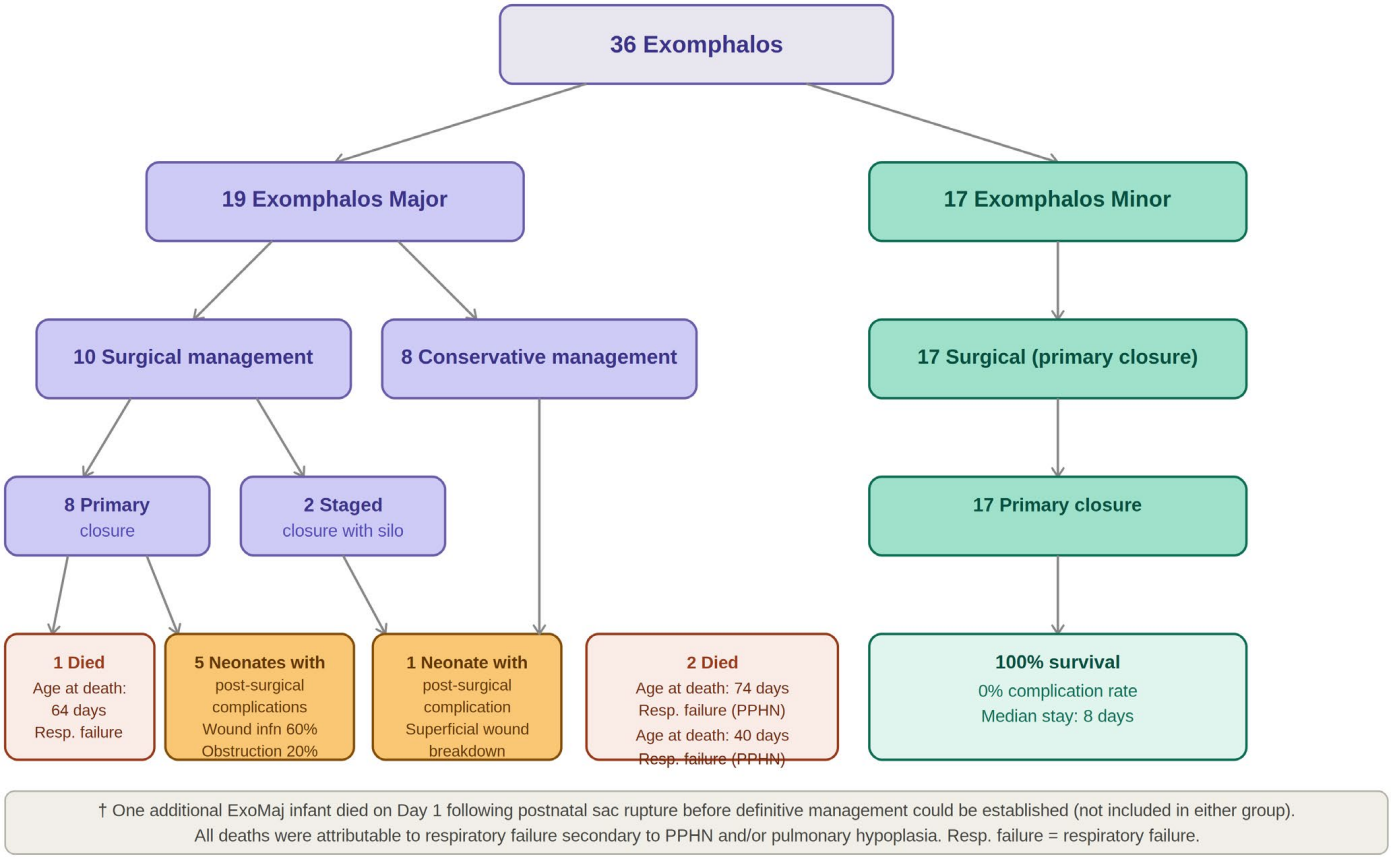
Flow diagram illustrating management pathways and outcomes for ExoMaj and ExoMin neonates, including post‐surgical complications and causes of death.

Surgically managed ExoMaj neonates had a significantly longer median hospital stay (24.5 vs. 8 days; *p* = 0.001), greater age to achieve full enteral feeds (20 vs. 7 days; *p* = 0.003), and a markedly higher complication rate (70% vs. 0%; *p* = 0.004) compared with ExoMin. ExoMin exhibited a significantly lower complication rate following primary closure than ExoMaj (0% vs. 75%; *p* = 0.001). Median age at first surgical intervention was 4 days (range 1–12) in ExoMaj and 3 days (range 1–8) in ExoMin (*p* = 0.345). Mortality in the ExoMaj surgical group was 1/10 (10%) compared with 0/17 (0%) in ExoMin (*p* = 0.370). No significant differences were identified in age at first surgical intervention or mortality between surgical groups. Conservative management was employed in 8/19 ExoMaj neonates (42%). One additional ExoMaj infant died on Day 1 following postnatal sac rupture before definitive management could be established. Comparing surgically and conservatively managed ExoMaj infants, complications occurred significantly more frequently in the surgical group (6/10 [60%] vs. 0/8 [0%]; *p* = 0.013). No significant differences were observed in mortality (1/10 [10%] vs. 2/8 [25%]; *p* = 0.559), length of hospital stay (24.5 days [range 13–65] vs. 27.5 days [range 21–42]; *p* = 0.327), or age at full enteral feeds (20 days [range 6–26] vs. 18 days [range 7–38]; *p* = 0.859) (Table 5).

**TABLE 5:** Outcomes by management strategy in ExoMaj and ExoMin.

Outcome	ExoMaj surgical (*n* = 10)	ExoMaj conservative (*n* = 8)	ExoMin surgical (*n* = 17)	*p* (ExoMaj surgical vs. ExoMin surgical)
Hospital stay, days	24.5	27.5	8	0.001
Age at full enteral feed, days	20	18	7	0.003
Complications, *n* (%)	6 (60%)	0 (0%)	0 (0%)	0.004
Mortality, *n* (%)	1 (10%)	2 (25%)	0 (0%)	0.559
Within ExoMaj surgical subgroups				
Primary closure (*n* = 8): hospital stay, days	22			0.476
Staged closure (*n* = 2): hospital stay, days	32			
Primary closure: complications	6/8 (75%)			1.000
Staged closure: complications	1/2 (50%)			

*Note:* Data are presented as median unless otherwise stated. No statistically significant differences were identified between ExoMaj surgical and conservative management, or between ExoMaj primary and staged closure. ExoMin: all 17 neonates underwent primary closure within 8 days; 0% complication rate and 100% survival.

Abbreviation: ns, not significant.

Wound infection (culture‐positive or documented purulence) was the most common postoperative complication in surgically managed ExoMaj (60%). Additional complications included post‐operative intestinal obstruction (20%), post‐operative feeding difficulties due to recurrent abdominal distension (20%), and incisional ventral hernia (20%).

Two ExoMaj neonates presented with postnatal sac rupture. One underwent staged closure via silo and experienced subsequent superficial wound breakdown. The other had a severe defect with a ruptured sac and virtually no residual skin; this infant was managed conservatively with cling film coverage of the internal organs and died on Day 1 of life. One ExoMaj neonate who underwent skin‐only closure subsequently required elective hernia repair. All ExoMin neonates underwent full fascial closure without prosthetic patch.

ExoMaj neonates undergoing staged closure (*n* = 2) had a median hospital stay of 30 days (range 29–31) and median age at full enteral feeds of 23.5 days (range 21–26), compared with 19 days (range 13–65) and 17 days respectively for primary closure (*n* = 8). Although ExoMaj neonates undergoing staged closure had numerically longer hospital stay and time to full feeds compared with primary closure, and conservatively managed ExoMaj had a numerically higher mortality than those managed surgically, none of these differences reached statistical significance, likely reflecting the small subgroup sizes. Follow‐up records confirmed that of the eight conservatively managed ExoMaj infants: two died, three subsequently underwent elective hernia repair via a “paint and wait” approach, and three are awaiting planned surgical closure.

## 4 Discussion

This 12‐year retrospective series provides contemporary outcome data for live‐born exomphalos neonates at a UK tertiary centre, and highlights three notable observations. First, all observed mortality was attributable exclusively to pulmonary complications, suggesting that respiratory disease—rather than associated anomalies per se—may represent an important contributor to adverse outcome in ExoMaj. Second, the type of associated anomaly differed markedly between ExoMaj and ExoMin, whilst overall anomaly burden did not, suggesting that anomaly type rather than presence may be the more clinically relevant variable. Third, conservative management was associated with acceptable outcomes in selected ExoMaj cases, consistent with the growing evidence base for the “paint and wait” approach. The overall mortality of 11% compares favorably with previously reported rates of 15%–25% [2, 3, 4], reflecting advances in neonatal intensive care, ventilatory support, and surgical technique.

### 4.1 Mortality and Pulmonary Complications

All four deaths occurred in ExoMaj neonates and were attributable to respiratory failure secondary to PPHN and/or pulmonary hypoplasia, consistent with Sakonidou et al. [2] and broader literature identifying pulmonary complications as a potential contributor to mortality in ExoMaj [5]. Whilst the small number of deaths precludes definitive conclusions, the consistency of this finding across all fatal cases strengthens the clinical plausibility of this association. Ages at death ranged from 1 to 74 days, reflecting the variable severity and clinical trajectory of pulmonary disease. The mechanical sequelae of ExoMaj—including diaphragmatic compression and elevated diaphragm position—further compromise respiratory function [6]. The presence of liver within the exomphalos sac and requirement for mechanical ventilation at birth are established predictors of PPHN [7], both prevalent in this cohort.

These observations highlight the potential importance of prenatal pulmonary assessment in ExoMaj, and suggest that optimizing respiratory management from birth—including early pulmonary vasodilator therapy and judicious ventilatory strategies—should be a central priority within the multidisciplinary care plan. The concurrent presence of structural cardiac lesions, particularly those causing left‐to‐right shunting such as PDA and VSD, may further exacerbate pulmonary hypertension by increasing pulmonary blood flow and pressure. In the context of pre‐existing pulmonary hypoplasia, this interaction may potentiate the severity of PPHN. Three of the four non‐survivors had concurrent cardiac lesions, highlighting the potential importance of detailed cardiac assessment alongside pulmonary evaluation in ExoMaj neonates.

### 4.2 Surgical vs. Conservative Management

Surgically managed ExoMaj infants experienced longer hospital stays, delayed full enteral feeds, and significantly higher complication rates compared with ExoMin, consistent with findings reported by Rijhwani et al. [8]. Wound infection was the most frequent complication (60%), alongside post‐operative intestinal obstruction, post‐operative feeding difficulties due to recurrent abdominal distension, and incisional hernia (each 20%). Importantly, no significant differences in any outcome measure were found between surgically and conservatively managed ExoMaj neonates. Conservative management was utilized in 8/19 ExoMaj neonates (42%) and demonstrated acceptable outcomes in six of these eight cases. Review of individual case records identifies three principal indications: (1) large intact sac with significant liver herniation deemed to confer prohibitive risk of primary closure due to anticipated abdominal compartment syndrome; (2) haemodynamic instability or significant pulmonary hypertension precluding safe operative intervention; and (3) severity of associated anomalies rendering surgical intervention futile, where conservative management was palliative. Our institutional protocol employed topical agents—silver sulfadiazine (Flamazine; Smith & Nephew, London, UK) or medical‐grade honey (Medihoney; Derma Sciences, Trenton, NJ)—to promote eschar formation with a view to delayed closure. The absence of significant differences in mortality, hospital stay, and time to full feeds between surgical and conservative groups, combined with the significantly lower complication rate in conservatively managed infants (0% vs. 60%; *p* = 0.013), supports further prospective evaluation of conservative management as a potential strategy for selected ExoMaj cases. These observations are consistent with the systematic review by Bauman et al. [9, 10, 11], who described nonoperative management with topical silver followed by delayed fascial closure as one approach for giant omphalocele. Our protocol incorporated both silver sulfadiazine (Flamazine (Smith & Nephew, London, UK)) and Medihoney (Derma Sciences, Trenton, NJ) dressings. None of our conservatively treated neonates proceeded to structured immediate delayed surgical closure, though three ultimately underwent elective hernia repair and three await closure—a trajectory consistent with the “paint and wait” philosophy described in the literature [9].

### 4.3 Associated Congenital Anomalies

Associated anomalies were present in 67% of the cohort, consistent with prior reports [1, 3]. A particularly noteworthy and, to our knowledge, under‐reported observation is that the overall incidence of associated anomalies did not differ significantly between ExoMaj and ExoMin, yet outcomes diverged markedly. This dissociation raises the hypothesis that it may be the type and severity of anomalies—rather than their mere presence—that influences outcomes; a finding with direct relevance to antenatal counseling and risk stratification. The predominance of pulmonary defects in ExoMaj (37%) and Beckwith‐Wiedemann syndrome in ExoMin (41%) mirrors findings by Malhotra et al. [5] and Groves et al. [12], and supports the concept of distinct anomaly predilections by exomphalos classification.

Correspondingly, no statistically significant differences in outcomes were identified between isolated and non‐isolated exomphalos in this series, though the study was not powered to exclude clinically meaningful differences. These observations suggest that prenatal counseling may benefit from considering not merely whether anomalies are present, but their specific nature, though this requires validation in larger studies. Families of ExoMaj infants should be specifically counseled about the risk of pulmonary complications and their implications for respiratory management, whilst those with ExoMin should be informed of the relatively high incidence of Beckwith‐Wiedemann syndrome and its associated surveillance requirements.

The low incidence of chromosomal anomalies in this live‐born cohort likely reflects selective termination following prenatal diagnosis, as previously noted [3].

## 5 Conclusion

In this 12‐year single‐centre cohort, infants with exomphalos major experienced greater morbidity and accounted for all mortality observed within the study population. Pulmonary complications were present in all fatal cases and represented an important clinical feature of exomphalos major in our cohort. Management strategies varied according to defect characteristics and clinical status, with both operative and conservative approaches utilized. Given the retrospective design, small sample size, and potential confounding factors, these findings should be considered descriptive and hypothesis‐generating. Our findings suggest that the nature of associated anomalies may be more clinically relevant than their presence alone, although confirmation in larger cohorts is required. Larger multicentre studies are required to define independent predictors of outcome and optimize management strategies.

This study has several limitations. The retrospective single‐centre design and small sample size (*n* = 36) restrict statistical power and limit generalisability. As only live‐born infants were included, outcomes may not reflect the complete population of fetuses diagnosed prenatally with exomphalos, particularly those with severe chromosomal abnormalities who may have undergone termination of pregnancy. Multivariable modeling was not performed as only four deaths occurred, rendering regression analyses statistically inappropriate. Selection bias may have influenced management allocation between surgical and conservative groups, as decisions were based on clinical judgment rather than a prospective protocol. Future multicentre prospective studies with standardized management protocols and longer follow‐up are needed to provide definitive evidence to guide clinical practice.

## Author Contributions

A.G.: data acquisition and analysis. C.K.S.: conception and design of the study, critical revision of the manuscript. S.S.: conception and design of the study, drafting the manuscript, critical revision. All authors read and approved the final manuscript.

## Funding

The authors have nothing to report.

## Conflicts of Interest

The authors declare no conflicts of interest.

## Data Availability

The data that support the findings of this study are available from the corresponding author upon reasonable request.
